# Characterisation of immune responses and protective efficacy in mice after immunisation with Rift Valley Fever virus cDNA constructs

**DOI:** 10.1186/1743-422X-6-6

**Published:** 2009-01-17

**Authors:** Nina Lagerqvist, Jonas Näslund, Åke Lundkvist, Michèle Bouloy, Clas Ahlm, Göran Bucht

**Affiliations:** 1 Swedish Defence Research Agency, Department of CBRN Defence and Security, SE-901 82 Umeå, Sweden; 2 Department of Clinical Microbiology, Division of Infectious Diseases, Umeå University, SE-901 85 Umeå, Sweden; 3 Department of Clinical Microbiology, Division of Virology, Umeå University, SE-901 85 Umeå, Sweden; 4 Swedish Institute for Infectious Disease Control, SE-171 82 Solna, Sweden; 5 Institut Pasteur, Unité de Génétique Moléculaire des Bunyaviridés, Paris, France; 6 National Environment Agency, Environmental Health Institute, 11 Biopolis Way, 06-05/08, Helios Block, 138667, Singapore

## Abstract

**Background:**

Affecting both livestock and humans, Rift Valley Fever is considered as one of the most important viral zoonoses in Africa. However, no licensed vaccines or effective treatments are yet available for human use. Naked DNA vaccines are an interesting approach since the virus is highly infectious and existing attenuated Rift Valley Fever virus vaccine strains display adverse effects in animal trials. In this study, gene-gun immunisations with cDNA encoding structural proteins of the Rift Valley Fever virus were evaluated in mice. The induced immune responses were analysed for the ability to protect mice against virus challenge.

**Results:**

Immunisation with cDNA encoding the nucleocapsid protein induced strong humoral and lymphocyte proliferative immune responses, and virus neutralising antibodies were acquired after vaccination with cDNA encoding the glycoproteins. Even though complete protection was not achieved by genetic immunisation, four out of eight, and five out of eight mice vaccinated with cDNA encoding the nucleocapsid protein or the glycoproteins, respectively, displayed no clinical signs of infection after challenge. In contrast, all fourteen control animals displayed clinical manifestations of Rift Valley Fever after challenge.

**Conclusion:**

The appearance of Rift Valley Fever associated clinical signs were significantly decreased among the DNA vaccinated mice and further adjustment of this strategy may result in full protection against Rift Valley Fever.

## Background

Rift Valley Fever virus (RVFV) is a mosquito-borne *Phlebovirus *in the *Bunyaviridae *family. RVFV infects domesticated ruminants and humans and regularly induces epizootics with concomitant epidemics throughout the African continent and on the Arabian Peninsula [1,2]. Outbreaks among domesticated ruminants are characterised by a large increase of spontaneous abortions and the case fatality rate may reach 100% in young animals [3]. While Rift Valley Fever (RVF) is generally benign in man, more severe clinical manifestations such as hemorrhagic fever, encephalitis and retinitis are regulary observed [4].

Despite the fact that RVF is an important viral zoonosis, and the risk for emergence in new susceptible areas has been emphasized [1], effective and safe vaccines are not commercially available. However, formalin inactivated vaccines have been developed for human use, but the distribution is limited to high-risk occupation staff [5,6]. Currently there are a few vaccines available for use in livestock: vaccines based on the live-attenuated Smithburn strain [7] and formalin inactivated virus preparations [8]. The Smithburn virus vaccine is suggested to induce lifelong protection, but has retained the ability to induce abortions and teratogenic effects in livestock [9,10]. The inactivated virus vaccines are safe, but less immunogenic and require annual booster vaccinations [11]. Previously, two vaccine candidates have been proposed and tested for their safety and efficacy in animal trials: a naturally attenuated RVFV isolate from a benign human case in the Central African Republic, Clone 13 [12] and a human virus isolate of RVFV attenuated in cell culture by 5-fluorouracil treatment, MP12 [13,14]. Although Clone 13 and MP12 were shown to be safe and immunogenic in mice and in cattle and sheep, respectively [12], the MP12 vaccine was found teratogenic for pregnant sheep if used during the first trimester [15].

In addition to the adverse effects previously shown for attenuated RVF vaccines, there are considerable safety concerns regarding viral vaccines based on highly pathogenic organisms due to the risk for exposure or escape of live agents during the manufacturing process. In addition, there is also a risk of insufficient inactivation or emergence of revertants, when large quantities of virulent virus strains are handled. Because of these shortcomings, new RVF vaccine strategies ought to be considered. Genetic immunisation is an attractive alternative, since the antigens are produced by the host cells and the presentation resembles natural infections by intracellular parasites. It is also cost-effective and circumvents the need for elevated biosafety level facilities [16]. Genetic vaccines are also less vulnerable to elevated temperatures during storage and transportation, which are important factors when performing vaccinations in developing countries [17]. These characteristics make DNA vaccines uniquely suited for vaccine production against highly pathogenic organisms, such as RVFV [18,19].

The RVFV is a three segmented negative stranded RNA virus. The (L)arge segment encodes a RNA dependent RNA polymerase and the (M)edium segment encodes two glycoproteins (G_N _and G_C_), a 78 kDa protein as well as a non-structural protein (NSm). The (S)mall segment encodes a non-structural protein (NSs) and the immunogenic and highly expressed nucleocapsid protein (N) [3].

Despite an abundance of the N protein in the virus and in the infected cell, this protein is not generally associated with protective immunity. However, a recent study has shown that a proportion of mice inoculated with purified RVFV N proteins were protected against virus challenge [20]. Although antibodies targeting the RVFV glycoproteins are recognized for their protective properties [21] contradictory results regarding the level of protection after DNA vaccination have been presented [20,22,23].

In this study we evaluate the induced immune responses and the conferred protection in mice after genetic immunisation with cDNA encoding the structural proteins of RVFV. The elicited immune responses towards the N, G_N_, G_C _and G_N_/G_C _proteins after gene-gun immunisation were analysed and the protective abilities of the N and the G_N_/G_C _construct were tested by virus challenge.

## Methods

### Cells and viruses

BHK-21 (ATCC number CCL-10) cells were maintained in Glasgow MEM (GIBCO, Invitrogen, Carlsbad, CA) supplemented with 5% FCS, 1.3 g/l Tryptose (Difco™, Becton, Dickinson and Company, Sparks, MD), 10 mM HEPES, 1 mM sodium pyruvate, 100 U penicillin/ml and 100 μg/ml streptomycin at 37°C/5% CO_2_. The working stocks of RVFV and cDNA constructs, originated from the ZH548 wild-type strain, isolated from a human case in Egypt in 1977 [24]. Viral stocks were prepared and titrated on monolayers of BHK-21 cells and the cDNA sequences are found under the GenBank accession numbers AF134534 and DQ380206[25,26].

### Production of DNA vaccine

For genetic immunisation and eukaryotic expression, cDNAs encoding N, G_N_/G_C_, G_N _and G_C _were inserted into pcDNA3.1/V5-His^® ^TOPO (Invitrogen). The primer sequences used for cDNA amplification and subsequent cloning are shown in Table 1. The correctness of each cDNA construct was confirmed by sequencing (MWG-Biotech) and the corresponding gene products were verified through transfection of mammalian cells followed by immunofluorescence analysis. A cDNA construct (pcDNA3.1) encoding the N protein (PUU-N) of the Puumala hantavirus (Puumala virus Umeå/hu [GenBank: AY526219] [27,28] was used as a control. The preparation of gene-gun cartridges has previously been described [28]. Briefly, 50 μg aliquots of the above plasmid DNA preparations were precipitated on 25 mg of 1 μm gold beads and subsequently used to coat the inner wall of Tefzel tubings according to the manufacturer's instructions (BioRad Laboratories, Hercules, CA). Each gene-gun cartridge delivered approximately 1 μg of DNA.

**Table 1:** Primers sequences

**Construct**	**Forward primer sequences**	**Reverse primer sequences**
^*a*^G_N_/G_C_	5'-ATGGAAGACCCCCATCTCAGAAA-3'	5'-CTATGAGGCCTTCTTAGTGGC-3'
^*a*^G_N_	5'-ATGGAAGACCCCCATCTCAGAAA-3'	5'-TGCTGATGCATATGAGACAATC-3'
^*a*^G_C_	5'-ATGTGTTCAGAACTGATTCAGGCA-3'	5'-CTATGAGGCCTTCTTAGTGGC-3'
^*a*^N	5'-CACCATGGACAACTATCAAGAGCTT-3'	5'-GGCTGCTGTCTTGTAAGCC-3'
^*a*^PUU-N	5'-CACCATGAGTGACTTGACAGATATCCA-3'	5'-TATCTTAAGTGGATCCTGATTAGATA-3'
^*b*^N	5'-CACCATGGACAACTATCAAGAGCTT-3'	5'-GGCTGCTGTCTTGTAAGCC-3'
^*b*^N1	5'-CACCATGGACAACTATCAAGAGCTT-3'	5'-ATCCCGGGAAGGATTCCCT-3'
^*b*^N2	5'-CACCATGATGATGAAAATGTCGAAAG-3'	5'-TTAAGAGTGAGCATCTAATATT-3'
^*b*^N3	5'-CACCATGCCGAGGCATATGATGCACC-3'	5'-GGCTGCTGTCTTGTAAGCC-3'
^*b*^N1/2	5'-CACCATGGACAACTATCAAGAGCTT-3'	5'-AGAGTGAGCATCTAATATT-3'
^*b*^N2/3	5'-CACCATGATGATGAAAATGTCGAAAG-3'	5'-TAAGGCTGCTGTCTTGTAAGCC-3'

^*a *^Eukaryotic expression.

^*b *^Prokaryotic expression.

### Animal immunisation and infection

Female BALB/c mice, six to eight weeks old, were used in this study. Before immunisation the mice were thoroughly shaved on the abdomen and vaccinated with cDNA encoding the antigens using a gene-gun (Helios™, BioRad Laboratories). The cDNA was administrated four times with two to three week intervals. The primary immunisation was performed using four gene-gun cartridges and the following three boosters with two cartridges. Blood samples were collected three, five, seven and nine weeks after the primary immunisation. In order to study the immune responses post infection (p.i.) and the effectiveness of the genetic vaccines, mice were injected intraperitoneally (i.p.) with RVFV diluted in sterile PBS to a final volume of 100 μl. Infected animals were kept in micro-isolator cages inside an animal isolator (Bell Isolation Systems Ltd, Livingston, Scotland) and all manipulations involving infected animals or viable virus were performed within a BSL-3 laboratory. During the experimental procedures the animals were monitored daily and were kept with free access to food and water. Mice found in a moribund condition (fatigue and "hunchback-like posture") were instantly euthanized. This project was approved by The Animal Research Ethics Committee of Umeå University, Sweden.

### Evaluation of immune response

To evaluate and compare the immune responses after vaccination and infection, eight animals were vaccinated with cDNA encoding N, four with cDNA containing the open reading frame of the G_N_/G_C _poly-protein and two groups, each containing four animals, were immunised with either the G_N _or the G_C _construct. To analyse the immune responses after infection, one group consisting of nine mice were infected with 2.4 × 10^4 ^PFU of RVFV. At day 14 p.i. the animals were euthanized and samples collected. As negative controls, four mice were immunised with the pcDNA3.1 vector without insert and another four mice were injected with sterile PBS and kept under the same conditions.

### Challenge study

A total of 30 mice were used in the challenge study, eight of which were vaccinated with cDNA encoding the RVFV N protein and eight with the G_N_/G_C _construct. As controls, eight animals were vaccinated with an irrelevant gene (encoding the N protein of the Puumala virus, PUU-N) and six animals with pcDNA 3.1 vectors without insert. After four rounds of immunisations, half of the mice of each vaccination group were challenged with 2.4 × 10^3 ^and half with 2.4 × 10^4 ^PFU of RVFV. Blood samples were collected every alternate day until the end of the experiment at day 17 p.i.

### Antigen production and purification

For antigen production and prokaryotic expression, cDNA encoding the full-length N protein (aa 1–245) of RVFV was ligated into pET-14b (Novagen, Darmstadt, Germany) and cDNA encoding truncated N derivatives, N1 (aa 1–100), N2 (aa 71–170), N3 (aa 141–245), N1/2 (aa 1–170) and N2/3 (aa 71–245), were inserted into pET101/D-TOPO^® ^or pET151/D TOPO^® ^(Invitrogen). The primer sequences are shown in Table 1.

DNA constructs expressing the N protein and truncated N derivatives were expressed in *Escherichia coli *(*E. coli*) BL21 DE3 (Invitrogen). Briefly, transformed bacteria were grown in Luria-Bertani media supplemented with 100 μg/ml carbencillin to OD A_600 _of 0.7. Expression of the antigens was induced by the addition of isopropyl-beta-D-thiogalactopyranoside (IPTG) at a final concentration of 0.5 mM. The purification of the full length N protein expressed from a poly-histidine-fusion vector was performed with metal chelating chromatography using Ni-NTA Agarose (Qiagen GmbH, Hilden, Germany), essentially as described previously [29]. N protein preparations used for the lymphocyte proliferation assay were purified further with Triton X-114 (Sigma-Aldrich Inc., St. Louis, MO) to remove contaminating amounts of endotoxins [30]. Each batch was tested for unspecific stimulation of splenocytes before use.

### Enzyme-linked immunosorbent assay (ELISA), Western blot and Immunofluorescence analysis (IFA)

Indirect ELISA (total Ig) was performed using microtiter plates (NUNC-immuno™ MaxiSorp, Nalgene Nunc International, Rochester, NY) coated with 3 μg/ml of purified recombinant N protein as previously described [31]. Wells lacking the primary antibody were used to establish the background levels and negative or pre-immune sera were used to determine unspecific binding.

Western blot was performed using *E. coli *extracts containing the complete N protein or truncated variants thereof (N1, N2, N3, N1/2, N2/3). The separated proteins were transferred to Immobilon TMP transfer membranes (type PVDF, Millipore Co., USA). Membranes containing the antigens were incubated with serum samples from individual mice at dilution 1:600 in parallel with internal controls, either an anti-V5 antibody (Invitrogen) diluted 1:5000 or a mouse anti-poly-histidine antibody (ZYMED^® ^Laboratories, S. San Francisco, CA) diluted 1:3000. A horseradish peroxidase (HRP) conjugated rabbit anti-mouse Ig antibody (DacoCytomation, Glostrup, Denmark) diluted 1:2000 was used as secondary antibody. The antibody-antigen complexes were visualised with enhanced chemiluminescence (ECL, Amersham Bioscience, Uppsala, Sweden). The blotting and incubation procedures have previously been described in detail [28].

For IFA, BHK-21 cells were grown on cover slips and infected with ZH548 at MOI 1, or transfected with cDNA constructs using FuGene™ reagent according to the manufacturer's instructions (Roche Diagnostics, Basel, Switzerland). At 36 h p.i. or 48 h post transfection the cells were fixed with 3% paraformaldehyde in PBS (for anti-glycoprotein antibody detection) or methanol (for anti-N antibody detection). Labelling was performed with mouse sera diluted 1:200, followed by visualisation with an Alexa Fluor™ 488 (Molecular probes, Invitrogen) secondary antibody at dilution 1:5000. The expression of the antigens was verified using an anti-V5 antibody (Invitrogen) diluted 1:5000, positive sera from previously infected mice or monoclonal antibodies directed against the G_N _and G_C _proteins, kindly provided by Dr. George Ludwig (USAMRIID, Fort Detrick, MD) at predetermined dilutions.

### Lymphocyte proliferation test

The lymphocyte proliferation assay was performed as described earlier [32]. Briefly, spleen cells of five mice vaccinated with cDNA encoding the full length N protein of RVFV were prepared in RPMI 1640 (GIBCO, Invitrogen) supplemented with 5% FCS, 2 mM sodium pyruvat, 2.5 × 10^-5 ^M β-Mercaptoethanol and 50 μg/ml gentamicin sulphate. After washing the spleen cells three times in cell culture media by centrifugation at 600 × g, the lymphocytes were resuspended to 4 × 10^5 ^cells/ml. Aliquots (100 μl) of the cells were seeded to 96-wells flat-bottom microplates (Nalgene Nunc International) in cell culture media containing the antigen at different concentrations. After two days incubation at 37°C/5% CO_2_, 1 μCi of ^3^HTdR (5'-^3^H Thymidine spec.act 14.4 Ci/mmol, Amersham Biosciences) was added. After an additional 16–18 hr of metabolic labelling, the cells were harvested on GF/C filters (Inotech AG, Basle, Switzerland) and analysed for incorporated radioactivity using a liquid scintillation counter (TriCarb 2500 TR, Packard Instruments, Meriden, CT). Spleen cells obtained from four mice immunised with the plasmid vector without insert constituted the negative control. The stimulation index (SI) was calculated as the ratio of radioactivity incorporated into cells from vaccinated mice and the count rate in cells from control mice.

### Plaque reduction neutralisation test (PRNT)

Heat-inactivated mouse sera including positive and negative controls, were serially diluted three-fold in PBS and incubated with a virus suspension containing about 30 plaque forming units (PFU) of RVFV. The mixtures were incubated for 90 min at 37°C and thereafter used to infect monolayers of BHK-21 cells in 6-well tissue culture plates (NUNC tissue culture, Nalgene Nunc International). After an adsorption period of 30 min at 37°C, the cells were rinsed with PBS and incubated with cell culture media containing 1% Carboxy-Methyl Cellulose (Aquacide II, Calbiochem^®^, Merck, CA) for six days at 37°C/5%CO_2_. The cells were subsequently fixed with 10% formaldehyde, washed with water and counter-stained with 1% crystal violet in water containing 20% ethanol and 0.7% NaCl. The PRNT_50 _titer was calculated as the reciprocal of the highest serum dilution that reduced the number of plaques by 50%, as compared to the virus control.

### Statistical methods

The outcome of the challenge was evaluated using the Fisher exact test (Epi Info™, Version 3.5). Quantitative variables were based on measurements of at least two independent experiments containing duplicate samples. Variables are expressed as means and the error bars represent the standard deviation.

## Results

### Antibody response after immunisation with cDNA encoding the N protein

**Figure 1:**
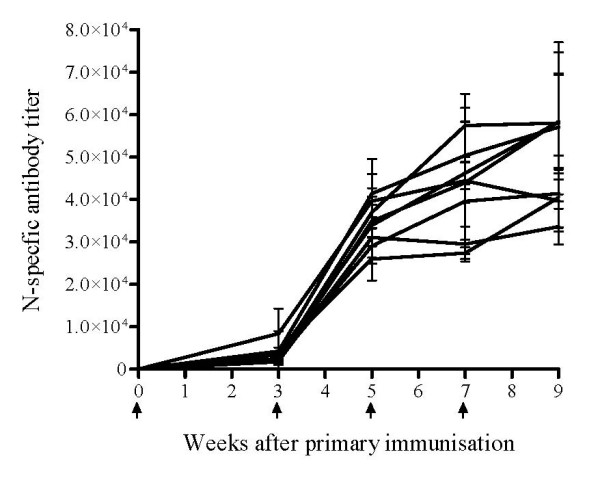
**Anti-N specific antibody responses (total Ig) after gene-gun vaccination with cDNA encoding the RVFV N protein**. The curves correspond to the mean titers in individual mouse sera measured by ELISA. The error bars represent the standard deviation between replicates. Arrows along the X-axis illustrate the time points of vaccination.

Genetic vaccination with cDNA encoding the N protein resulted in a strong humoral immune response in all mice. Anti-N specific antibodies (total Ig) were detected by ELISA already after the first immunisation and were followed by a large increase in titers after additional vaccination rounds (Fig 1). However, despite the strong antibody response observed after genetic vaccination with cDNA encoding the N protein, RVFV neutralising antibodies were not detected by PRNT (data not shown).

**Figure 2:**
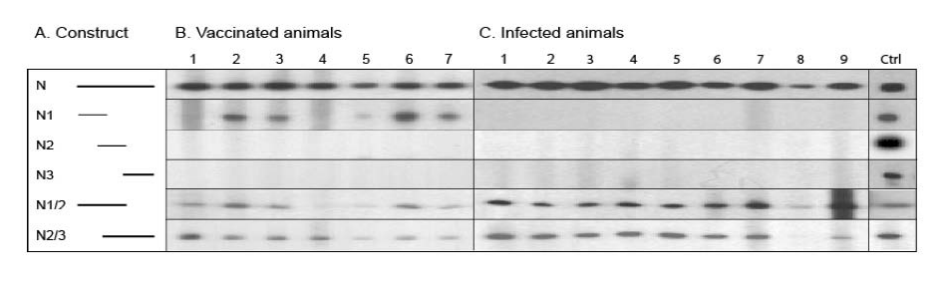
**Western blot reactivity towards the N protein and truncated variants thereof**. (A) Schematic presentation of the full length and deleted variants of the RVFV N antigens. Different filter strips represent different recombinant proteins. The sera were obtained from (B) seven mice vaccinated with cDNA encoding the complete N protein or (C) nine mice infected with RVFV. The sera were collected after four immunisations or 14 days p.i., respectively. Antibodies binding to the amino- or carboxy-terminal His-tag or V5-tag of the recombinant proteins were used as positive controls (Ctrl).

Since previous studies have shown that strong antigenic determinants are located near the amino-terminus of the N protein of other viruses in the *Bunyaviridae *family [33,34], antigenic regions of the RVFV N protein were investigated in more detail. Serum samples from seven mice immunised with cDNA encoding the complete N protein and nine from infected mice were analysed and compared by Western blot for reactivity towards the N protein and truncated N proteins (Fig 2). A strong and uniform reactivity profile towards the full-length protein was found in all animals and most sera displayed a similar but weaker reactivity towards the truncated N1/2 and N2/3 proteins. Surprisingly, the amino-terminus (N1 protein) was only recognised by sera from immunised mice and not by any serum obtained from infected mice. Furthermore, the central part (N2) and the carboxy-terminus (N3) were neither recognised by sera from infected nor immunised mice (Fig 2).

### Proliferative response subsequent immunisation with cDNA encoding the N protein

**Figure 3:**
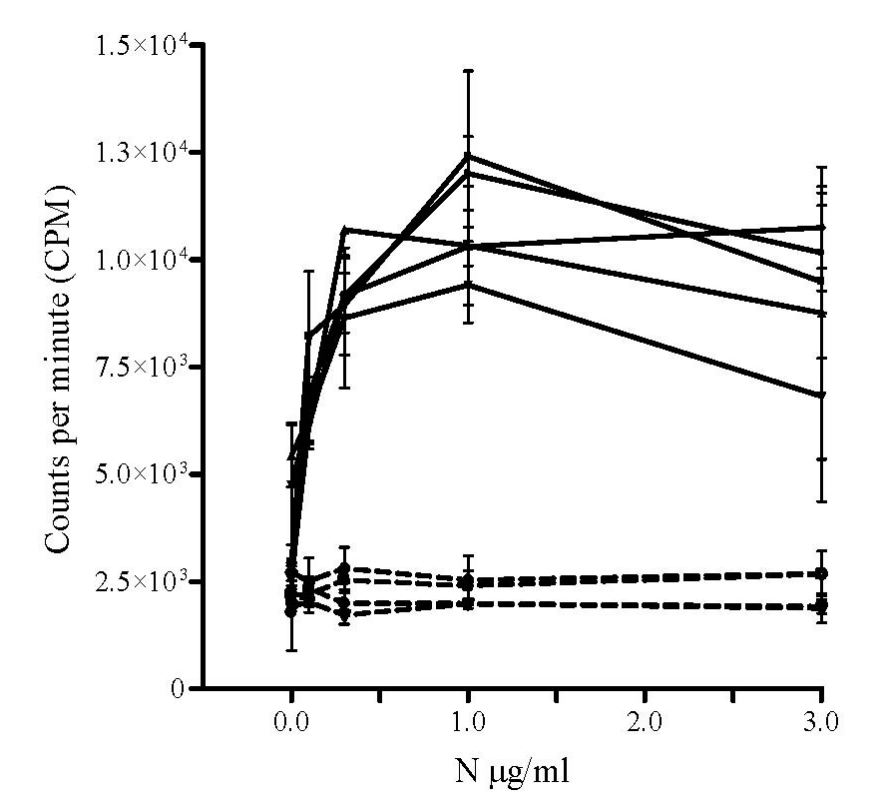
**Lymphocyte proliferation test performed on spleen cells from mice vaccinated with cDNA encoding the N protein**. The curves correspond to the incorporated radioactivity measured for cells of five immunised mice and the dotted curves represent four control mice immunised with the vector without insert. The error bars represent the standard deviation between replicates. The spleen cells were stimulated for proliferation using the indicated N antigen concentrations (0.1, 0.3, 1.0 and 3.0 μg/ml).

Spleen cells from vaccinated mice were assayed to address the question if genetic immunisation induces antigen dependent cell proliferation. The obtained results indicate that lymphocytes from five animals immunised with the N construct displayed antigen induced proliferation when up to 1 μg/ml of the purified and Triton X-114 extracted N protein was added (Fig 3). However, higher concentrations of the antigen (5–10 μg/ml) resulted in cell toxicity and cell death. The stimulation index (SI) was determined at between 4 and 6 when spleen cells were stimulated with 1 μg/ml of the purified N antigen (Fig 3). Background levels, independent of the antigen concentration, were observed in lymphocytes from control mice. Incorporated radioactivity in spleen cells stimulated by 0.5–1 μg of ConA was approximately 10–20 times higher that of the negative controls and 4–5 times higher than any cell sample collected from immunised mice.

### Humoral response after immunisation with cDNA encoding the glycoproteins

All mice sero-converted after immunisation with cDNA encoding the G_N_/G_C _proteins or the G_N _protein but only two out of four after vaccination with cDNA encoding the G_C _protein, as detected by IFA performed on infected cells. The virus neutralising antibody titers after G_C _and G_N _vaccination were in the lower range, less than 25 and between 25 to 75, respectively. However, the G_N_/G_C _vaccinated mice acquired considerably higher titers, up to 225 (data not shown). These results indicate that vaccination with the G_N_/G_C _construct resulted in higher virus neutralising antibody titers than the use of cDNA encoding for the individual glycoproteins.

### Challenge of gene-gun vaccinated mice

To evaluate the degree of protection against RVFV infection after gene-gun vaccination, a new batch of mice was divided into groups of eight and immunised with either cDNA encoding the N or the G_N_/G_C _proteins. Two control groups, eight mice immunised with the PUU-N construct and six mice immunised with vectors without insert were also included. The groups were further divided into two subgroups and challenged with 2.4 × 10^3 ^or 2.4 × 10^4 ^PFU of RVFV (Table 2). As the lethality of the ZH548 strain was found low for the 15 to 17 weeks old BALB/c mice, the protection conferred by vaccination was also based on development of clinical signs and increase in N specific antibody titers (the latter was only applied for G_N_/G_C _vaccinated mice) upon challenge. In the G_N_/G_C _vaccination group, all mice responded to the vaccination and sero-converted, while only five out of eight mice developed virus neutralising titers ranging from 25 to 75 (Table 2). Mice vaccinated with the N construct induced a strong antibody response, with ELISA titers ranging from 2.5 × 10^4 ^to 4.5 × 10^4^, after four immunisations (data not shown).

**Table 2:** Neutralising antibody titers and outcome after challenge after DNA vaccination against RVFV

**Vaccine group**	**No. of animals**	**PRNT_50 _titers^*a*^**	**Challenge dose**	**Outcome after challenge with RVFV**		
				Asymptomatic	Clinical signs^*b*^	Deaths^*c*^

N	4	< 25	2.4 × 10^3^	1	3	

	4	< 25	2.4 × 10^4^	3		1

G_N_/G_C_	4	25 – 75	2.4 × 10^3^	2	2	

	4	25 – 75	2.4 × 10^4^	3	1	

Ctrl/PUU-N	4	-	2.4 × 10^3^		3	1

	4	-	2.4 × 10^4^		3	1

Ctrl/pcDNA3.1	3	-	2.4 × 10^3^		3	

	3	-	2.4 × 10^4^		3	

^*a *^Virus neutralising antibody titers after vaccination.

^*b *^Number of animals displaying clinical signs (ruffled fur/shivering), followed by complete recovery.

^*c *^Number of animals displaying a moribund condition (fatigue/"hunchback-like posture") followed by euthanization.

Since differences in clinical signs could not be ascribed to the different challenge doses, the two subgroups within each vaccine group were consolidated and evaluated together. In the groups of mice immunised with the N or the G_N_/G_C _constructs, four of eight and five of eight animals, respectively, displayed no clinical signs during the entire experiment (Table 2). Despite the large proportion of animals without RVF clinical signs in the G_N_/G_C _vaccination group, extensive viral replication after infection was indicated by high N specific antibody titers, similar to the titers observed for the control animals (data not shown). Apart from one casualty, due to a moribund condition, in the N vaccinated group, no major differences in the severity of the clinical manifestations were observed between the G_N_/G_C _and N vaccinated mice after challenge. In contrast, all animals in the two control groups displayed either clinical signs of infection followed by complete recovery (12/14) or were sacrificed due to a moribund condition (2/14) (Table 2). Significant protection against RVF clinical signs was observed among the N vaccinated mice (*p *= 0.0096, Fisher exact test) and the G_N_/G_C _vaccinated mice (*p *= 0.0021, Fisher exact test) as compared to the controls.

## Discussion

RVF is an important emerging zoonotic infection and early efforts to protect animals and humans resulted in development of attenuated and inactivated virus vaccines. Vaccines based on live attenuated RVFV strains have shown to induce long-lasting protection in contrast to inactivated virus vaccines, which require multiple booster doses to retain a protective immunity [11]. Unfortunately, teratogenic effects and the ability to cause abortions limit the likelihood for wide use and distribution of the current vaccines based on attenuated RVFV strains. As the existing vaccines have such shortcomings, efforts to design safer and more efficient RVF vaccines need to be undertaken.

We have investigated the prospect of employing genetic immunisation against RVF. The DNA vaccine platform has been extensively studied during the last decade. However, the breakthrough has been on halt until recently when the first licensed products became available, such as the vaccine against West Nile virus infection in horses and a vaccine for use in salmon against the hematopoietic necrosis virus [35]. The DNA vaccine technology is especially suitable against pathogens such as RVFV, since the need of elevated biosafety facilities are circumvented and the stability of these vaccines allow distribution in developing countries lacking the logistics to maintain a "cold-chain".

In this study, the immune responses in mice after genetic immunisation with RVFV cDNA encoding the N protein, the glycopolyprotein G_N_/G_C_, and the separate G_C _and G_N _proteins were analysed. The N and the G_N_/G_C _constructs displayed the most promising results regarding the elicited immune response and were evaluated further for the ability to confer protection in a subsequent challenge study.

After gene-gun vaccination with the N construct, high antibody titers were repeatedly induced along with an antigen induced proliferative cellular response. Interestingly, no clinical signs were observed after challenge in 50% of the animals (compared to 100% in the control group) despite the lack of detectable levels of neutralising antibodies after vaccination. The observed protection might be explained by cell-mediated immune factors as indicated by the dose-dependent proliferation of spleen cells from the immunised animals. Nevertheless, the characteristics of the proliferating cells remain to be investigated further. Analogous results were previously found after vaccination with the purified RVFV N protein when protection was obtained in 60% of the vaccinated mice [20]. Also, a recent study using the Toscana virus (*Phlebovirus, Bunyaviridae*) reported approximately 60% survival upon challenge after immunisation with the recombinant N protein, probably due to a cellular mediated immune response [36].

Previous studies of N proteins of Hantaviruses revealed that strong B-cells epitopes are located near the amino-terminus [33,34]. However, this does not seem to be the case for RVFV N. Genetic immunisations are in general believed to mimic the natural presentation of antigens [37], but interestingly, while the sera of immunised mice recognized the amino-terminal part (aa 1–100) of the N-protein, sera of the infected animals did not. The lack of reactivity towards the central (N2) and the C-terminal (N3) parts could either be explained by a distorted conformation of the encoded antigens or disruption of epitope-regions within the N protein.

In this study, antibodies towards the glycoproteins were induced after genetic vaccination, but virus neutralisation was only observed in sera of mice immunised with cDNA containing the G_N _gene. This observation is in accordance with earlier findings, where G_N _has been shown to possess antigenic determinants important for protection, while G_C _does not [38,39]. However Besselar and co-workers found neutralising epitopes associated with protection in the G_C_, as well as in the G_N _protein [40]. The absence of neutralising antibodies after gene-gun vaccination using the G_C _construct alone might be explained by incorrect folding of the expressed antigen, since neutralising antibodies elicited by the glycoproteins are often found to be conformation dependent [41].

The RVFV glycoproteins have been used in several protection studies, utilizing different vaccination strategies and animal models. The protective effect varied from no/low to complete protection depending on the administration strategy, antigen and animal model used [20-22,38-40,42]. In this study, the majority of the G_N_/G_C _vaccinated mice were protected against RVF. However, the incomplete protection found was unexpected as a similar study, using analogous G_N_/G_C _constructs (RVFV_-NSm_), reported complete protection of mice after challenge [22]. On the other hand, intramuscular inoculation of cDNA encoding the G_N_/G_C _polyprotein did not induce neutralising antibodies and did not protect against RVFV challenge [20]. Interestingly, a recent study reported that dual expression of the N and the G_N_/G_C _proteins may generate RVF Virus-Like Particles (VLPs) [43], and the formation of VLPs after genetic immunisation is hypothesised to be the reason for the high virus neutralising antibody titers induced by the genetic West Nile virus vaccine [44]. Perhaps, by using a similar approach, and introducing cDNA encoding the N and the G_N_/G_C _proteins of RVFV, a fully protective immune response might be induced.

In summary, while DNA vaccination against RVF induced strong humoral and proliferative immune responses in vaccinated mice, complete protection after challenge was not achieved. Nevertheless, naked DNA vaccines may constitute a promising strategy for vaccine development and this study provides insight for the basis of a future development of an efficacious DNA vaccine against RVF.

## Competing interests

The authors declare that they have no competing interests.

## Authors' contributions

NL made the cDNA constructs, carried out the serological assays, analysed the data and wrote the manuscript. JN carried out the vaccinations and challenge, performed the neutralisation tests and wrote the manuscript. ÅL has critically revised the manuscript and the experimental design. MB made contributions to the initial stages of conceiving the study and provided important intellectual content. CA helped in designing the experiments and in the writing of the manuscript. GB conceived of the study, designed and coordinated the research and drafted the manuscript. All authors read and approved the final manuscript.
